# Landscapes of SARS-CoV-2-reactive CD8^+^ T cells: heterogeneity of host immune responses against SARS-CoV-2

**DOI:** 10.1038/s41392-021-00589-1

**Published:** 2021-04-09

**Authors:** June-Young Koh, Eui-Cheol Shin

**Affiliations:** 1 Graduate School of Medical Science and Engineering, Korea Advanced Institute of Science and Technology (KAIST), Daejeon, Republic of Korea; 2 The Center for Epidemic Preparedness, KAIST, Daejeon, Republic of Korea

**Keywords:** Adaptive immunity, Infectious diseases

In a recent study published in *Science Immunology*,^1^ Kusnadi et al. performed single-cell RNA sequencing (scRNA-seq) of SARS-CoV-2-reactive CD8^+^ T cells and reported heterogeneity.

SARS-CoV-2 infection causes COVID-19, which is an ongoing pandemic disease threatening public health. The virology of SARS-CoV-2 and immune responses against the virus have been urgently investigated to develop effective measures against COVID-19. During viral infection, CD8^+^ T cells contribute to elimination of the virus by exerting cytotoxicity against virus-infected cells and producing effector cytokines, whereas neutralizing antibodies interfere with viral entry of host cells.

After the emergence of COVID-19, early studies examined the phenotypes and functions of various subtypes of immune cells from infected patients using high-dimensional techniques, including scRNA-seq and multi-parameter cytometry.^2,3^ These studies also revealed the profiles of CD8^+^ and CD4^+^ T cells in patients with COVID-19. However, the data did not include information regarding virus-specificity of T cells because these studies analyzed total CD8^+^ or CD4^+^ T cells, not SARS-CoV-2-reactive CD8^+^ or CD4^+^ T cells.

Other studies have detected and characterized SARS-CoV-2-reactive CD8^+^ and CD4^+^ T cells using ex vivo antigen stimulation-based assays, including interferon (IFN)-γ ELISpot assays, intracellular cytokine staining (ICS), and activation-induced marker (AIM) assays.^4^ Intriguingly, SARS-CoV-2-reactive CD8^+^ and CD4^+^ T cells have been detected not only in COVID-19 patients and convalescents, but also unexposed individuals. MHC class I (MHC-I) multimers were also used to directly detect SARS-CoV-2-specific CD8^+^ T cells without ex vivo stimulation, and their phenotypes were examined among COVID-19 patients and convalescents.^5^ Although these studies examined the phenotypes and functions of SARS-CoV-2-reactive T cells, high-dimensional techniques, such as scRNA-seq, could not be combined; thus, the deep profiles of SARS-CoV-2-reactive T cells have not been elucidated.

**Fig. 1:**
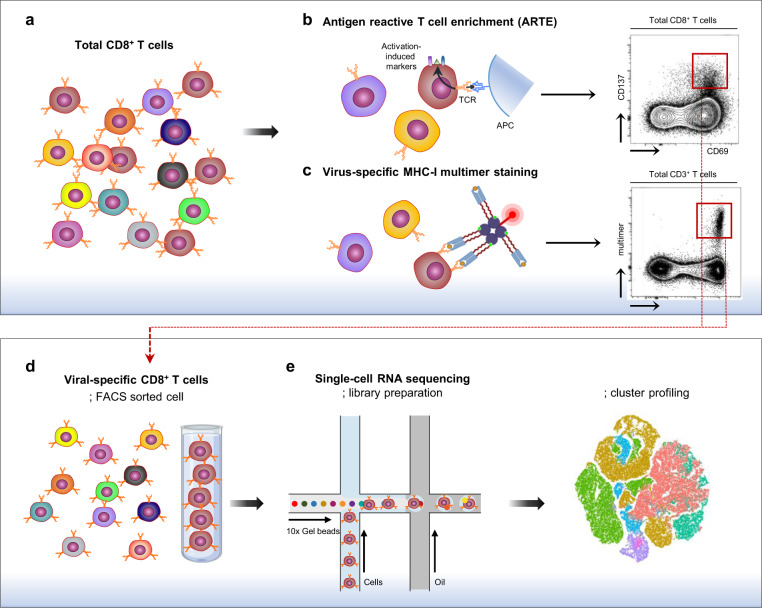
Single-cell RNA sequencing of virus-specific CD8^+^ T cells. **a** Total CD8^+^ T cells include CD8^+^ T cells with various antigen specificity. **b**, **c** Virus-specific CD8^+^ T cells are fluorescently detected by activation-induced markers, such as CD69 and CD137, following ex vivo stimulation with viral antigens (**b**) or MHC-I multimer staining (**c**). **d** Virus-specific CD8^+^ T cells are enriched by sorting fluorescently stained cells. The procedure enriching activation-induced marker^+^ cells is called antigen-reactive T-cell enrichment (ARTE). **e** Enriched virus-specific CD8^+^ T cells are analyzed by single-cell RNA sequencing, and single-cell heterogeneity is revealed

In a recent study, Kusnadi et al. examined landscapes of the SARS-CoV-2-reactive CD8^+^ T-cell population in a comparison with influenza A virus (IAV)-reactive and respiratory syncytial virus (RSV)-reactive CD8^+^ T-cell populations by scRNA-seq analysis.^1^ First, they isolated each virus-reactive CD8^+^ T-cell population from the peripheral blood mononuclear cells (PBMCs) of patients with COVID-19, or healthy donors via modified antigen-reactive T-cell enrichment (ARTE) (Fig. 1). In modified ARTE, PBMCs were stimulated ex vivo for 24 h with overlapping peptide pools for each viral protein, and responding CD8^+^ T cells were isolated based on the expression of activation markers CD137 and CD69. Next, they performed scRNA-seq analysis of each viral protein-reactive CD8^+^ T-cell population.

They analyzed the single-cell transcriptome and T cell receptor (TCR) sequence of >84,000 virus-reactive CD8^+^ T cells from 49 subjects in total, including patients with COVID-19 and healthy donors. Virus-reactive CD8^+^ T cells created seven clusters according to gene expression profiles, indicating heterogeneity among virus-reactive CD8^+^ T cells. They then described distinct characteristics of SARS-CoV-2-reactive CD8^+^ T cells compared to IAV-reactive or RSV-reactive CD8^+^ T cells. SARS-CoV-2-reactive CD8^+^ T cells from patients with COVID-19 and healthy donors were mainly composed of clusters enriched with T-cell exhaustion signature genes, IFN-stimulated genes, and cytotoxicity-related genes. In contrast, IAV-reactive or RSV-reactive CD8^+^ T cells were mainly composed of clusters enriched with inflammatory cytokine genes. They concluded that SARS-CoV-2-reactive CD8^+^ T cells exhibit exhausted phenotypes with type I IFN stimulation, and have a decreased capacity to secrete inflammatory cytokines.

Focusing on the transcriptome and TCR sequence data of SARS-CoV-2-reactive CD8^+^ T cells from patients with mild and severe COVID-19, they attempted to differentiate mild and severe COVID-19. SARS-CoV-2-reactive CD8^+^ T cells from patients with severe COVID-19 had a significantly lower frequency of the exhausted cluster than mild patients. When the analysis was narrowed down to the exhausted cluster, severe COVID-19-specific upregulated genes were highly enriched with cytotoxicity-related genes, pro-inflammatory cytokine genes, and genes for T-cell activation-associated transcription factors and negatively enriched with IFN response genes. These findings suggest that SARS-CoV-2-reactive CD8^+^ T cells are less exhausted, and more functional with an impaired type I IFN response in severe compared to mild COVID-19.

They also analyzed the non-exhausted cluster. Severe COVID-19-specific upregulated genes were enriched with genes related to co-stimulation and NF-κB activation, suggesting that SARS-CoV-2-reactive CD8^+^ T cells are more activated in patients with severe disease than those with mild disease. In the analysis of TCR clonality, clonal expansion was increased in SARS-CoV-2-reactive CD8^+^ T cells from patients with severe disease compared to those with mild disease. Collectively, SARS-CoV-2-reactive CD8^+^ T cells present a robust response in severe patients.

Kusnadi et al. reported a valuable resource for understanding the heterogeneity of the host immune response against SARS-CoV-2 infection by investigating SARS-CoV-2 reactive CD8^+^ T cells with the modified ARTE assay and scRNA-seq analysis. Unlike previous studies investigating total CD8^+^ T cells, this study described a landscape of SARS-CoV-2-reactive CD8^+^ T cells isolated by modified ARTE for the first time.

ARTE is a useful technique for enriching T cells reactive to specific antigens. However, it has inherent limitations for the proper characterization of antigen-reactive CD8^+^ T cells. Because the process for ARTE includes ex vivo stimulation of T cells with overlapping peptide antigens, the phenotypes and transcriptomes of antigen-reactive T cells can be changed by stimulation. In addition, ARTE cannot capture antigen-specific, non-functioning T cells. This is critical because a considerable proportion of antigen-specific CD8^+^ T cells detected by MHC-I multimer staining do not exert effector functions.^5^ These limitations can be overcome by using DNA barcode-tagged MHC-I multimers in scRNA-seq analysis (Fig. 1). MHC-I multimer staining enables the detection of virus-specific CD8^+^ T cells without stimulation regardless of their functions. However, MHC-I multimer combined scRNA-seq analysis has not yet been reported in the study of SARS-CoV-2-specific CD8^+^ T cells in patients with COVID-19.

One of the main findings by Kusnadi et al. is that SARS-CoV-2-reactive CD8^+^ T cells are mainly clustered in the exhausted subset. However, a recent study demonstrated that, among SARS-CoV-2-specific CD8^+^ T cells detected by MHC-I multimer staining, PD-1^+^ cells, as well as PD-1^−^ cells, produced IFN-γ in patients with COVID-19 regardless of disease severity, indicating that SARS-CoV-2-specific CD8^+^ T cells are not exhausted, but functional.^5^ Further studies are required to examine the functional characteristics of CD8^+^ T cells in the exhausted cluster identified by Kusnadi et al. In addition, further studies are required to reveal a possible association between co-morbidities of COVID-19 patients and T cell functions.

The COVID-19 pandemic has urged us to investigate host immune responses, including the SARS-CoV-2-specific T-cell response. High-dimensional analysis adopting ARTE or MHC-I multimers will uncover the molecular characteristics, functions, and heterogeneity of SARS-CoV-2-specific CD8^+^ T cells in COVID-19 patients.
